# Nocardioides segetis sp. nov., isolated from the sandy soil of a long-term continuous cropping cotton field

**DOI:** 10.1099/ijsem.0.007160

**Published:** 2026-05-26

**Authors:** Delong Kong, Hao Xu, Siya Wu, Xinran Wang, Xu Jiang, Zhiyong Ruan, Wei Zhang

**Affiliations:** 1 Xinjiang Key Laboratory of Special Species Conservation and Regulatory Biology, College of Life Sciences, Xinjiang Normal University, Urumqi, 830054, PR China; 2 State Key Laboratory of Efficient Utilization of Arable Land in China, CAAS-CIAT Joint Laboratory in Advanced Technologies for Sustainable Agriculture, Institute of Agricultural Resources and Regional Planning, Chinese Academy of Agricultural Sciences, Beijing, 100081, PR China; 3 Technical Research Center for Environmental Geotechnical Engineering Restoration and Resource Utilization, Xinjiang Normal University, Urumqi 830054, PR China

**Keywords:** 16S rRNA gene, *Nocardioides*, polyphasic taxonomy

## Abstract

A Gram-stain positive, aerobic, rod-shaped bacterium, designated BYT-33-1^T^, was isolated from a typical sandy soil in Xinjiang, PR China. This isolate grew well at 20–40 °C, pH 6.0–10.0 and 0–3.0% (w/v) NaCl, with optimal growth at 30 °C, pH 7.0 and 0.5% NaCl, respectively. Phylogenetic analysis of 16S rRNA gene sequences indicated that BYT-33-1^T^ shared the highest sequence similarities with *Nocardioides nitrophenolicus* NSP 41^T^ (98.4%) and *Nocardioides kongjuensis* A2-4^T^ (98.3%). The draft genome was 5,327,936 bp, consisting of 24 contigs, with a G+C content of 72.4 mol%. The average nucleotide identity and digital DNA–DNA hybridization values between strain BYT-33-1^T^ and its closely related type strains of genus *Nocardioides* were 88.1–80.5% and 35.0–23.5%, respectively. The predominant menaquinone was MK-8(H_4_), and C_18:0_ 10-methyl and iso-C_16:0_ constituted the major cellular fatty acids. The major polar lipids were diphosphatidylglycerol and phosphatidylglycerol, one unidentified aminolipid (AL) and three unidentified phospholipids. The ll-diaminopimelic acid was the diagnostic diamino acid in the cell-wall peptidoglycan. In addition, strain BYT-33-1^T^ exhibits hexadecane degradation ability, suggesting its potential application value in alkane bioremediation of contaminated environments. Based on chemotaxonomic, phylogenetic, phenotypic and genomic analyses, we propose a novel species, named *Nocardioides segetis* sp. nov., with the type strain BYT-33-1^T^ (=CCTCC AA 2024001^T^=KCTC 59242^T^).

## Introduction

The genus *Nocardioides* of the family *Nocardioidaceae* was first described by Prauser *et al*. [1]. At the time of writing, 178 species with validly published names in the genus *Nocardioides* are listed (https://lpsn.dsmz.de/genus/nocardioides) [2]. Members of the genus *Nocardioides* are Gram-stain positive, rod- or coccus-shaped, diphosphatidylglycerol (DPG), phosphatidylglycerol (PG) and phosphatidylinositol as the major polar lipids, and ʟʟ-diaminopimelic acid as the diagnostic diamino acid in cell-wall peptidoglycan with MK-8(H_4_) as the major menaquinone. The DNA G+C content is between 67.5 and 74.9 mol% [3,4]. They have widespread distribution in environments such as soils, plastic waste, coastal sediment and wastewater [4,8]. Members of the genus *Nocardioides* exhibit versatile metabolic capabilities and show excellent potential in degrading a wide range of environmental pollutants, including alkanes, aromatic compounds and various pesticides. For example, *Nocardioides alcanivorans* NGK65^T^ for hexadecane degradation and *Nocardioides limicola* DJM-14^T^ for alkane degradation [9]. Other representative functional strains include ‘*Nocardioides carbamazepini*’ CBZ_1^T^ capable of ibuprofen degradation, *Nocardioides nitrophenolicus* NSP 41^T^ for *p*-nitrophenol degradation and *Nocardioides soli* mbc-2^T^ for carbendazim degradation [10]. In addition, some species possess special biosynthetic potential, such as *Nocardioides jiangxiensis* WY-20^T^, a novel actinobacterium isolated from lakeside soil exhibiting mycofactocin biosynthesis potential [11]. In this study, a novel strain designated BYT-33-1^T^ was isolated from a sandy soil sample collected from a cotton field. Preliminary tests indicated that this strain also has the ability to degrade alkanes and aromatic compounds. Based on genotypic, phylogenetic, phenotypic and chemotaxonomic characterization, the isolate is considered to represent a new species of the genus *Nocardioides*.

## Methods

### Isolation

Strain BYT-33-1^T^ was obtained from the sandy soil of a long-term continuous cropping cotton field in Wujiaqu area, Urumqi, Xinjiang (44° 17′ 24″ N 87° 55′ 39″ E), China. Serially diluted sterile water samples were spread on medium of Reasoner’s 2A agar (R2A; BD/Difco) and incubated at 30 °C for 48 h. A single colony was subcultured on R2A agar to obtain pure culture. The purified culture was maintained on R2A slants at 4 °C and as glycerol suspensions (25%, v/v) at −80 °C. The strains *N. nitrophenolicus* CGMCC 4.6873^T^, *Nocardioides kongjuensis* CGMCC 4.6877^T^ and *Nocardioides humi* CGMCC 4.6878^T^, obtained from the China General Microbiological Culture Collection Centre (CGMCC), were used as reference strains.

### 16S rRNA gene phylogeny

For 16S rRNA gene sequencing, the genomic DNA of the strain BYT-33-1^T^ was extracted using the method described by Weisburg *et al*. [12]. The PCR product was purified and ligated into the pMD19-T cloning vector (Takara) following the manufacturer’s instructions. The PCR products of 16S rRNA gene were sequenced by the Life Technologies Company (Haidian District, Beijing, PR China) with the primers 27F and 1492R and compared with available sequences by using the EzBioCloud database [13]. Phylogenetic trees were reconstructed according to the neighbour-joining (NJ) [14], maximum-likelihood (ML) [15] and maximum-parsimony (MP) [16] by using mega 12 [17]. Evolutionary distances were calculated according to the algorithm of the Kimura’s two-parameter model, and the bootstrap analysis was used to evaluate based on 1,000 replicates [18].

### Genome analysis

For draft genome sequencing and assembly, the genomic DNA of the strain BYT-33-1^T^ was collected according to the method of Sun *et al*. [19]. The genomic G+C content of strain BYT-33-1^T^ was determined by whole-genome sequencing, which was performed on the Illumina MiSeq platform and assembled with SOAPdenovo. The completeness and contamination of the genome assembly were evaluated using the checkM software. The complete 16S rRNA gene sequence was extracted from the draft genome sequencing and compared with available sequences in the EzBioCloud database. Average nucleotide identity (ANI) and digital DNA–DNA hybridization (dDDH) values were calculated according to the proposed minimum standard for the reference of new species [20]. The ANI value between two genomes was calculated using the OrthoANIu algorithm (https://www.ezbiocloud.net/tools/ani). The *in silico* dDDH values between two strains were calculated by Genome-to-Genome Distance Calculator 2.1 (http://ggdc.dsmz.de/) [21]. On the basis of the genomic data of strain BYT-33-1^T^ and its related species, a whole-genome phylogeny was generated using the Type Strain Genome Server (TYGS; http://tygs.dsmz.de) [22]. Gene functional annotation analysis was performed using DIAMOND software to compare coding-protein gene sequences with Kyoto Encyclopedia of Genes and Genomes (KEGG), the Clusters of Orthologous Groups (COG) and Swiss-Prot databases [23,25].

### Phenotypic tests

Cell morphology in exponentially growing culture was examined by using a light microscope (Nikon 80i, Tokyo, Japan) and a transmission electron microscope (Hitachi 7500, Tokyo, Japan). Physiological characteristics were examined by growing the isolates on R2A medium under a variety of conditions. The temperature range for growth was determined by incubating strain BYT-33-1^T^ at 4, 10, 15, 20, 25, 30, 35, 40, 45 and 50 °C. The optimal concentration of NaCl for growth was investigated by using NaCl-free Luria–Bertani (LB; BD) broth with different NaCl concentrations (0, 0.5 and 1–7% at 1% increments, w/v). The pH range for growth was determined in LB broth that was adjusted to pH 4.0–12.0 at intervals of 0.5 pH units using sterile solutions of citric acid/Na_2_HPO_4_ (pH 4.0 to 5.0), Na_2_HPO_4_/NaH_2_PO_4_ buffer (pH 6.0 to 8.0), NaHCO_3_/Na_2_CO_3_ buffer (pH 9.0 to 10.0) or Na_2_HPO_4_/NaOH buffer (pH 11.0) or KCl/NaOH buffer (pH 12.0) [26]. Cell motility was examined by using the hanging-drop technique. Gram reaction, catalase and oxidase activities and hydrolysis of starch, tyrosine, carboxymethylcellulose, casein, gelatin and Tween 80 were detected by standard methods [27,28]. Additional physiological and biochemical features were determined using the API ZYM and 20NE strips (bioMérieux, L'Étoile, France) as described by the manufacturers’ instructions. To determine the application potential of strain BYT-33-1^T^, its ability to utilize hexadecane as a sole carbon source was tested in mineral salt medium (MSM). The MSM contained (per litre) NH_4_NO_3_ 1.0 g, KH_2_PO_4_ 1.0 g, Na_2_HPO_4_ 1.0 g, MgSO_4_·7H_2_O 0.2 g, CaCl_2_ 0.02 g, FeCl_3_ 0.001 g and pH 7.0. Hexadecane was sterilized by filtration and added at a final concentration of 0.2% (v/v) as the sole carbon source. Strain BYT-33-1^T^ was inoculated and incubated at 30 °C with shaking at 160 r.p.m. for 7 days, with all treatments performed in triplicate. After incubation, residual hexadecane was extracted with n-hexane, and the degradation rate was determined by gas chromatography with flame ionization detection [29].

### Chemotaxonomic analysis

Strains BYT-33-1^T^, *N. nitrophenolicus* CGMCC 4.6873^T^, *N. kongjuensis* CGMCC 4.6877^T^ and *N. humi* CGMCC 4.6878^T^ were harvested from cultures grown on tryptic soy agar medium at 30 °C. Fatty acid methyl esters were extracted and identified with the Sherlock Microbial Identification System (MIDI) with a GC (6890 N, Agilent) using the database RTSBA6 for identification of the peaks [30]. Cell-wall peptidoglycan was prepared and purified according to Schleifer and Kandler [31]. Quantitative analysis of peptidoglycan diamino acid was performed by GC as described [32]. Isoprenoid quinones were extracted from freeze-dried cells and analysed as described previously using LC-MS. Polar lipids of strain BYT-33-1^T^ were extracted and separated by two-dimensional TLC using silica gel 60 F 254 aluminium-backed thin-layer plates (Merck) [33]. The total lipids were revealed using molybdophosphoric acid hydrate ethanol solution. Phospholipids (PLs), glycolipids and ALs were determined using zinzadze reagent, *α*-naphthol reagent and ninhydrin reagent, respectively [34].

## Results and discussion

### 16S rRNA gene phylogeny

**Fig. 1.**
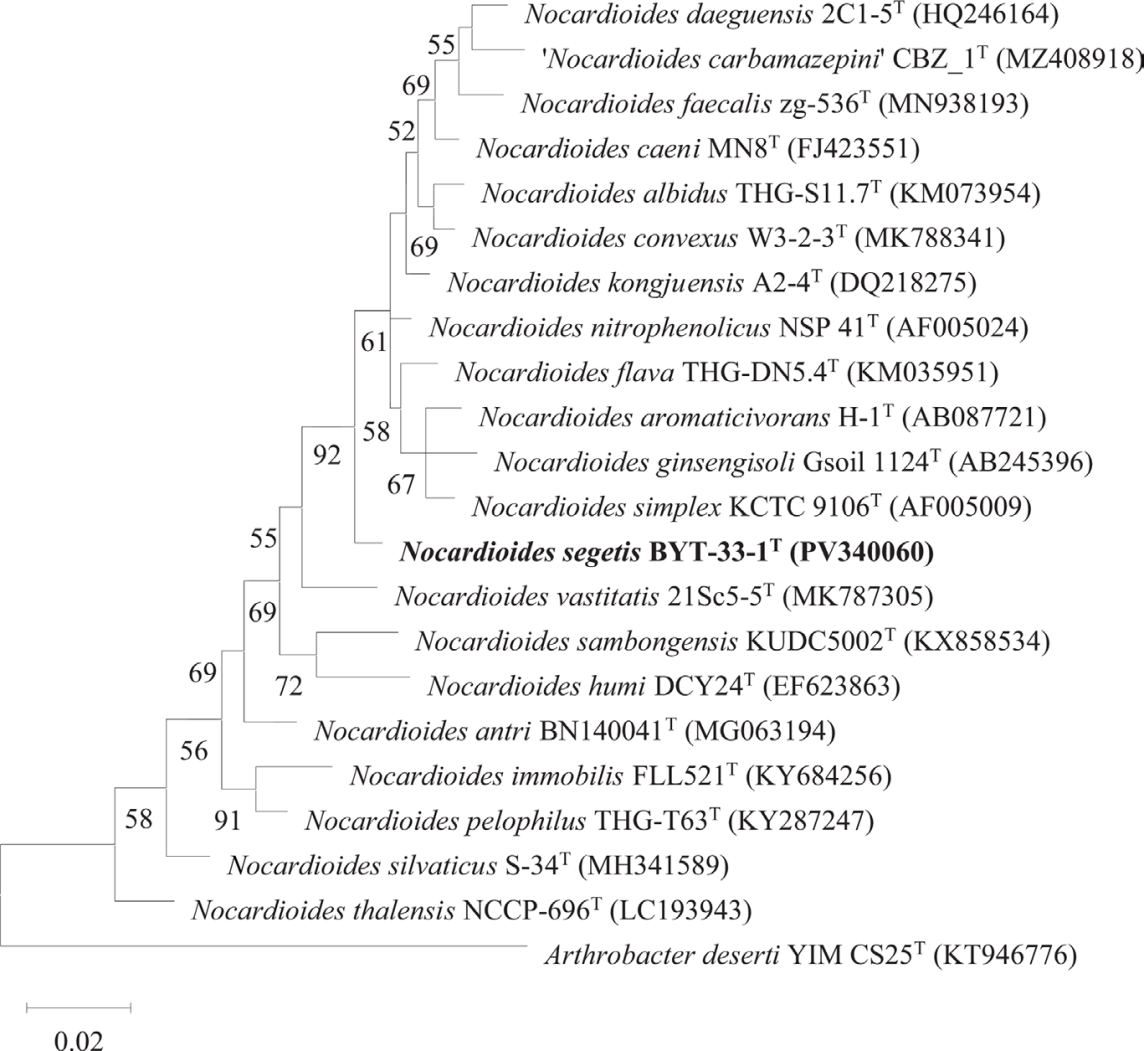
ML phylogenetic tree based on a comparison of the 16S rRNA gene sequences of strain BYT-33-1^T^ and its closest relatives. GenBank accession numbers are given in parentheses. Bar, 0.02 nucleotide changes per 1,000 nucleotides. Bootstrap values (>50%) based on 1,000 replications are shown at branch nodes.

The nearly complete 16S rRNA gene sequence of strain BYT-33-1^T^ (1,399 bp) was identical to the corresponding full-length sequence (1,519 bp) retrieved from its genome sequence. Comparative 16S rRNA gene sequence analysis revealed that the novel strain represents a member of the genus *Nocardioides*. The degree of sequence similarity for strain BYT-33-1^T^ was found to be with *N. nitrophenolicus* NSP 41^T^ (98.4%), *N. kongjuensis* A2-4^T^ (98.3%), *Nocardioides flava* THG-DN5.4^T^ (98.1%) and ‘*N. carbamazepini*’ CBZ_1^T^ (98.0%) and exhibited less than 98.0% sequence similarity with the type strains of other species with validly published names. Based on 16S rRNA gene sequences, the MP tree showed that strain BYT‑33‑1^T^ clustered with the type strains of *N. nitrophenolicus* NSP 41^T^ and *N. flava* THG‑DN5.4^T^ (Fig. S1, available in the online Supplementary Material); by contrast, the ML tree revealed that the strain formed a separate monophyletic branch (Fig. 1), and the NJ tree displayed an identical topology (Fig. S2). The results of phylogenetic tree analysis indicated that BYT-33-1^T^ represented a member of the genus *Nocardioides*.

### Genome characterization and phylogenomics

**Fig. 2.**
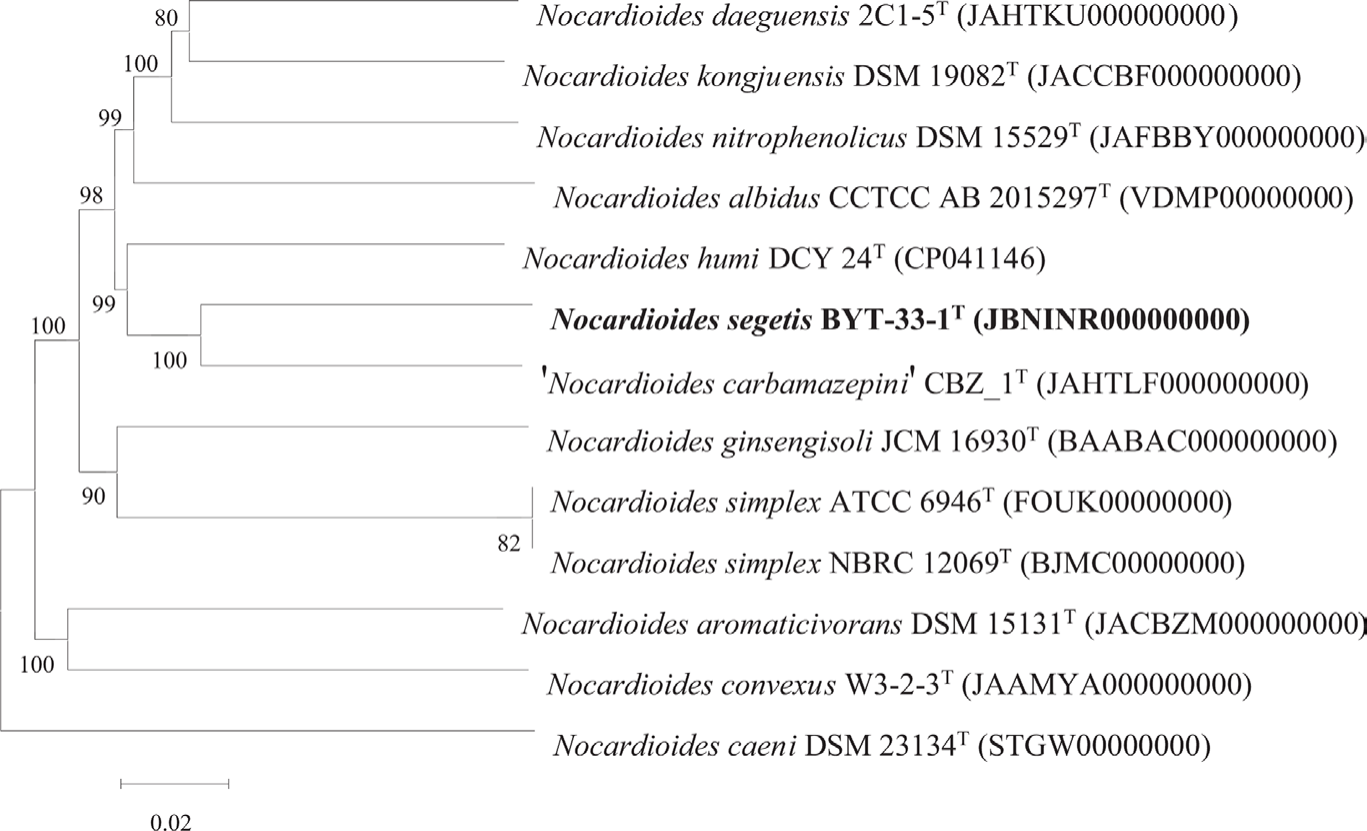
Phylogenomic tree of strain BYT-33-1^T^ and closely related strains based on core genomes was generated using TYGS. The tree was inferred with FastME from GBDP distances calculated from genome sequences. Genomes of closely related taxa were available in NCBI GenBank. The numbers above the branches are GBDP pseudo-bootstrap support values >50% from 100 replications. Bar 0.02 nucleotide substitutions per site.

The draft genome sequence of strain BYT-33-1^T^ was determined using the Illumina NovaSeq platform with a paired-end (2×150 bp) library with an insert size of 400 bp. The genome was 5,327,936 bp in size, consisting of 49 contigs, with an N20 of 498,034 bp and an N50 of 356,691 bp. Genome assembly was conducted with a k-mer size of 19 and an average sequencing depth of 278×. The DNA G+C content was 72.4 mol%, and the average sequencing depth was 967.54; raw read was 11,258,690 bp, and 49 tRNAs and 3 rRNAs were predicted for strain BYT-33-1^T^ (Fig. S3). CheckM analysis using the *Actinomycetales* marker lineage confirmed that the genome assembly showed 99.22% completeness and 0% strain heterogeneity. To verify the authenticity and purity of the genome data, the full-length 16S rRNA gene sequence was extracted from the draft genome assembly and showed 100% identity with the sequence obtained by PCR and Sanger sequencing. Genomic phylogenetic analysis showed that strain BYT-33-1^T^ belongs to the genus *Nocardioides* and clustered on one branch with ‘*N. carbamazepini*’ CBZ_1^T^ and *N. humi* DCY 24^T^ (Fig. 2). The genomic similarity between strain BYT-33-1^T^ and related strains was calculated. The OrthoANIu value between strain BYT-33-1^T^ and *N. nitrophenolicus* NSP 41^T^, *N. kongjuensis* A2-4^T^, ‘*N. carbamazepini*’ CBZ_1^T^ and *N. humi* DCY 24^T^ were 85.0%, 85.2%, 88.1% and 85.4%, respectively. The dDDH value between strain BYT-33-1^T^ and *N. nitrophenolicus* NSP 41^T^, *N. kongjuensis* A2-4^T^, ‘*N. carbamazepini*’ CBZ_1^T^ and *N. humi* DCY 24^T^ were 29.3%, 29.5%, 35.0% and 30.1%, respectively (Table S1). Based on the functional annotation against the COG database using eggnog-mapper, classification of the known proteins into functional categories revealed that transcription (449), amino acid transport and metabolism (435), lipid transport and metabolism (320), energy production and conversion (313), inorganic ion transport and metabolism (283) and secondary metabolites biosynthesis, transport and catabolism (281) were the most abundant COG categories (Fig. S4).

The genomic analysis of strain BYT-33-1^T^ revealed the presence of *alkB*, *alkA*, *alkJ*, *alkX*, *aldH*, *adh*, *lipA*, *lipB*, *fadA*, *fadB*, *fadD* and *fadE* genes related to alkane degradation (Table S2). Alkane hydroxylase encoded by the *alkB* gene is the most critical gene for alkane degradation [35,36]. The *aldH* and *adh* genes encode aldehyde dehydrogenase and alcohol dehydrogenase, which are functional enzymes in the downstream pathway of alkane degradation. Within the alkane degradation pathway of bacteria, *alkJ* and *alkX* serve as auxiliary factors involved in electron transport and transcriptional regulation, respectively. The *fadA*, *fadB*, *fadD* and *fadE* genes encode key enzymes involved in the *β*-oxidation pathway, responsible for the sequential degradation of fatty acid intermediates produced during alkane catabolism, while *lipA* and *lipB* encode enzymes that mediate the hydrolysis of ester bonds and further catabolism of alkane-derived intermediates [37,38]. In addition, genes encoding *kshA*, *kshB*, *hsaA*, *hsaB*, *hsaC* and *hsaD*, which participate in the aerobic catabolism of steroids and aromatic compounds, were identified in the genome. The products of *hsaA* and *hsaB* catalyse the formation of a catechol moiety, followed by extradiol cleavage catalysed by *hsaC* and hydrolytic cleavage catalysed by *hsaD*, representing typical steps in bacterial aromatic catabolism [39]. These degradation-related genes mainly reflect the ecological adaptation of strain BYT-33-1ᵀ to its hydrocarbon-containing environment, rather than providing taxonomically diagnostic characteristics for species differentiation.

### Phenotypic tests

Cells of strain BYT-33-1^T^ were aerobic, Gram-stain positive, non-motile and rod-shaped. The size of the cells was between 0.4 and 0.7 µm wide and 0.7 to 1.2 µm long (Fig. S5). The temperature range for growth was 20 °C to 40 °C (optimum: 30 °C), and pH range was 6.0 to 10.0 (optimum: 7.0). The NaCl concentration range for growth was from 0 to 3.0% (optimum: 0.5%). Strain BYT-33-1^T^ and the reference strain *N. nitrophenolicus* NSP 41^T^, *N. kongjuensis* A2-4^T^, ‘*N. carbamazepini*’ CBZ_1^T^ and *N. humi* DCY 24^T^ were positive for H_2_S production, hydrolysis of Tween 80, alkaline phosphatase, esterase (C4), esterase lipase (C8), leucine arylamidase, valine arylamidase, cystine arylamidase, trypsin, acid phosphatase and *α*-glucosidase. Strain BYT-33-1^T^ was negative for aesculin hydrolysis, *β*-galactosidase activity and glucose assimilation, but ‘*N. carbamazepini*’ CBZ_1^T^ and *N. humi* DCY 24^T^ were positive. Strain BYT-33-1^T^ was negative for starch hydrolysis and gluconate activity and mannitol assimilation, which was different from strains *N. nitrophenolicus* NSP 41^T^ and *N. kongjuensis* A2-4^T^. The distinctive physiological characteristics of strain BYT-33-1^T^ and its close phylogenetic relatives are given in Table 1. Furthermore, strain BYT-33-1^T^ could efficiently degrade hexadecane as the sole carbon source, with a degradation rate of 45.6% within 7 days, showing its potential application value in alkane bioremediation (Fig. S6). This combination of phenotypic traits, together with phylogenetic and genomic evidence, supports its classification as a novel species.

**Table 1.** Differential characteristics of strain BYT-33-1^T^ and its relative reference strains in the genus *Nocardioides* Strains: 1, BYT-33-1^T^; 2, *N. nitrophenolicus* CGMCC 4.6873^T^; 3, *N. kongjuensis* CGMCC 4.6877^T^; 4, *N. humi* CGMCC 4.6878^T^; 5, ‘*N. carbamazepini*’ CBZ_1^T^. Symbols: +, positive; −, negative; w, weak reaction.

Characteristic	1	2	3	4	5^*^
Growth temperature (°C)	20–40	15–40	10–40	25–42	15–37
pH range for growth	6–10	6–10	6–8	5–11	6–9
Growth in NaCl (%w/v)	0–3	0–3	0–5	0–3	0–3
Oxidase	+	+	+	+	−
Catalase	+	+	+	−	+
**Hydrolysis of:**					
Aesculin	−	+	+	+	+
Casein	−	−	+	−	+
Urea	−	+	−	−	−
Starch	−	+	+	+	−
**Enzyme activities (API ZYM):**					
Lipase (C14)	−	−	−	−	+
*α*-Chymotrypsin	−	−	−	+	−
Naphthol-AS-BI-phosphohydrolase	w	+	+	+	+
*β*-Galactosidase	−	w	w	+	+
*β*-Glucosidase	w	w	+	+	+
**Assimilation of (API 20NE):**					
Nitrate reduction	−	+	−	−	+
Glucose	−	−	+	+	+
Mannose	−	w	−	−	−
Mannitol	−	−	w	+	−
Gluconate	−	w	w	+	−
Adipate	−	−	w	+	−
Malate	+	w	w	+	+
Citrate	−	−	w	−	−
DNA G+C content (mol%)	72.4	71.4	72.1	71.0	71.4

*Data from Benedek *et al*. [8].

### Chemotaxonomic characterization

The major cellular fatty acids (>10%) of strain BYT-33-1^T^ contained C_18:0_ 10-methyl (23.4%) and iso-C_16:0_ (22.8%) that were similar to *N. nitrophenolicus* NSP 41^T^ and *N. kongjuensis* A2-4^T^, which were in accordance with the patterns observed in the type strains of *N. nitrophenolicus* NSP 41^T^ and *N. kongjuensis* A2-4^T^. The percentage of C_17:1_* ω6*c was lower than that of the reference strains. In addition, the content of C_18:1_* ω9*c was lower than that of the reference strain *N. nitrophenolicus* NSP 41^T^ (Table 2). The cell-wall peptidoglycan contained ll-diaminopimelic acid as a diagnostic diamino acid and MK-8(H_4_) as the only respiratory quinone detected in strain BYT-33-1^T^, consistent with other members of the genus *Nocardioides* [3,4]. Strain BYT-33-1^T^ contained DPG, PG and one unidentified PL, which was similar to the reference strain ‘*N. carbamazepini*’ CBZ_1^T^. In addition, one unidentified AL and two unidentified PLs (PL1–PL2) were also present as polar lipids (Fig. S7).

**Table 2.** Cellular fatty acid contents of strain BYT-33-1^T^ and its relative reference strains in the genus *Nocardioides* Strains: 1, BYT-33-1^T^; 2, *N. nitrophenolicus* NSP 41^T^; 3, *N. kongjuensis* A2-4^T^; 4, *N. humi* CGMCC 4.6878^T^. Values were percentages of total fatty acids. tr, trace amount (<0.5%).

Percentage				
Fatty acid	1	2	3	4
C_16:0_	5.6	2.7	2.5	4.4
C_17:0_	1.4	1.5	0.9	1.0
C_18:0_	5.3	0.8	4.8	3.3
C_16:0_ 2-OH	2.3	1.0	1.9	2.5
C_17:0_ 2-OH	0.6	0.7	tr	nd
C_17:0_ 10-methyl	3.7	4.4	5.7	3.1
C_18:0_ 10-methyl	23.4	17.6	13.6	20.7
C_17:1_* ω*6c	tr	7.4	12.6	8.0
C_17:1_* ω*8c	1.1	0.7	1.6	2.2
C_18:1_* ω*9c	8.9	12.1	9.2	15.9
Iso-C_15:0_	1.8	4.4	2.7	3.4
Iso-C_16:0_	22.8	26.0	23.5	10.4
Iso-C_16:1_ H	1.3	2.6	0.9	1.9
Iso-C_17:0_	8.1	5.9	4.5	9.7
Iso-C_18:0_	2.2	1.6	1.4	0.6
Anteiso-C_17:0_	2.4	3.1	4.8	2.0
Summed feature 3	2.4	0.9	1.1	nd
Summed feature 8	2.7	0.7	1.0	1.9
Summed feature 9	2.9	1.2	1.9	2.2

Summed features are fatty acids that cannot be resolved reliably from another fatty acid using the chromatographic conditions chosen. The MIDI system groups these fatty acids together as one feature with a single percentage of the total. Summed feature 3 includes C_16:1_* ω*6c and/or C_16:1_* ω*7c, summed feature 8 includes C_18:1_* ω*6c and/or C_18:1_* ω*7c and summed feature 9 includes C_16:0_ 10-methyl and/or iso-C_17:1_* ω*9c.

In summary, based on phylogenetic analysis and phenotypic and chemotaxonomic characteristics, strain BYT-33-1^T^ represents a new species of the genus *Nocardioides*, for which the name *Nocardioides segetis* sp. nov. is proposed.

## Description of *Nocardioides segetis* sp. nov.

*Nocardioides segetis* (se.geʹtis. L. gen. n. *segetis*, of the soil, referring to the isolation site).

Cells are Gram-stain positive, aerobic, non-motile, rod-shaped, 0.4–0.7 µm wide and 0.7–1.2 µm long. Colonies are circular, smooth, convex and pale yellow after 48 h growth on R2A at 30 °C. Grows at 20–40 °C (optimum: 30 °C), pH 6.0–10.0 (optimum: 7.0) and in the presence of 0–3% (w/v) NaCl (optimum: 0.5%). Catalase- and oxidase-positive. Gelatin and Tween 80 are hydrolysed and negative for hydrolysis of starch, casein, arginine, tyrosine and urea. In the API ZYM strip, positive results for alkaline phosphatase, esterase (C4), esterase lipase (C8), leucine arylamidase, valine arylamidase, cystine arylamidase, trypsin, acid phosphatase, *α*-glucosidase and *N*-acetyl-*β*-glucosaminidase, weak results for naphthol-AS-BI-phosphohydrolase and *β*-glucosidase and negative results for lipase (C14), *α*-chymotrypsin, *α*-galactosidase, *β*-galactosidase, *β*-glucuronidase, *α*-mannosidase and *α*-fucosidase. Strain BYT-33-1^T^ showed positive results for malate assimilation and negative for other reactions. Strain BYT-33-1^T^ contained MK-8(H_4_) as the respiratory quinone. The major cellular fatty acids are C_18:0_ 10-methyl and iso-C_16:0_. The ll-diaminopimelic acid was the diagnostic diamino acid in the cell-wall peptidoglycan. The main polar lipids are DPG and PG, one unidentified AL and three unidentified PLs.

The type strain is BYT-33-1^T^ (=CCTCC AA 2024001^T^=KCTC 59242^T^), which was isolated from sandy soil sample collected from Urumqi, Xinjiang, China. The DNA G+C content is 72.4 mol%. The accession number for the 16S rRNA gene sequence of strain BYT-33-1^T^ is PV340060. The GenBank/EMBL/DDBJ accession number for the draft genome sequence of strain BYT-33-1^T^ is JBNINR000000000.

## Supplementary material

10.1099/ijsem.0.007160 Uncited Supplementary Material 1.

## Abbreviations

ANI: average nucleotide identity
COG: Clusters of Orthologous Groups
dDDH: dgital DNA–DNA hybridization
DPG: diphosphatidylglycerol
ML: maximum-likelihood
MP: maximum-parsimony
MSM: mineral salt medium
NJ: neighbour-joining
PG: phosphatidylglycerol
R2A: Reasoner’s 2A
TYGS: Type Strain Genome Server
