# Supplementary material for: Nocardioides segetis sp. nov., isolated from the sandy soil of a long-term continuous cropping cotton field
Source: Int J Syst Evol Microbiol. 2026 May 26;76(5):007160. doi: 10.1099/ijsem.0.007160 (PMC13374528; doi:10.1099/ijsem.0.007160)

Nocardioides segetis sp. nov., isolated from the sandy soil of a long-term continuous cropping cotton field

Delong Kong, Hao Xu, Siya Wu, Xinran Wang, Xu Jiang, Zhiyong Ruan*, Wei Zhang*

Correspondence: Wei Zhang, 372981458@qq.com; Zhiyong Ruan, ruanzhiyong@caas.cn

Table. S1. The DDH and ANI values between strain BYT-33-1^T (NCBI accession number JBNINR000000000) and the related strains.

Strains	Number	G+C (%)	BYT-33-1^T	
			DDH (%)	ANI (%)
Nocardioides nitrophenolicus DSM 15529^T	JAFBBY000000000	72.49	29.3	85.0
Nocardioides kongjuensis DSM 19082^T	JACCBF000000000	71.84	29.5	85.2
'*Nocardioides carbamazepini*' CBZ_1^T	JAHTLF000000000	71.43	35.0	88.1
Nocardioides humi DCY24^T	CP041146	72.3	30.1	85.4
Nocardioides aromaticivorans DSM 15131^T	JACBZM000000000	71.91	25.3	82.1
Nocardioides simplex NBRC 12069^T	BJMC00000000	72.86	27.3	83.6
Nocardioides daeguensis 2C1-5^T	JAHTKU000000000	72.01	29.4	85.3
Nocardioides caeni DSM 23134^T	STGW00000000	71.27	23.5	80.5
Nocardioides convexus W3-2-3^T	JAAMYA000000000	72.04	24.6	81.7
Nocardioides albidus CCTCC AB 2015297^T	VDMP00000000	71.89	28.5	84.4
Nocardioides ginsengisoli JCM 16930^T	BAABAC000000000	72.0	26.6	83.2

Table. S2. Genes and metabolic modules of alkane degradation on the genome of strain BYT-33-1^{T}.

Enzyme (Gene)	Gene locus
alkB	scaffold9_3301
alkA	scaffold4_1793
alkJ	scaffold17_4338
alkX	scaffold9_3303
aldh	scaffold8_3116
aldh1	scaffold4_2100
adh	scaffold1_409
adh	scaffold1_575

Fig. S1. Maximum-parsimony phylogenetic tree of strain BYT-33-1^{T} and its relatives based on the comparison of the 16S rRNA gene sequences. Genbank accession numbers were given in parentheses.

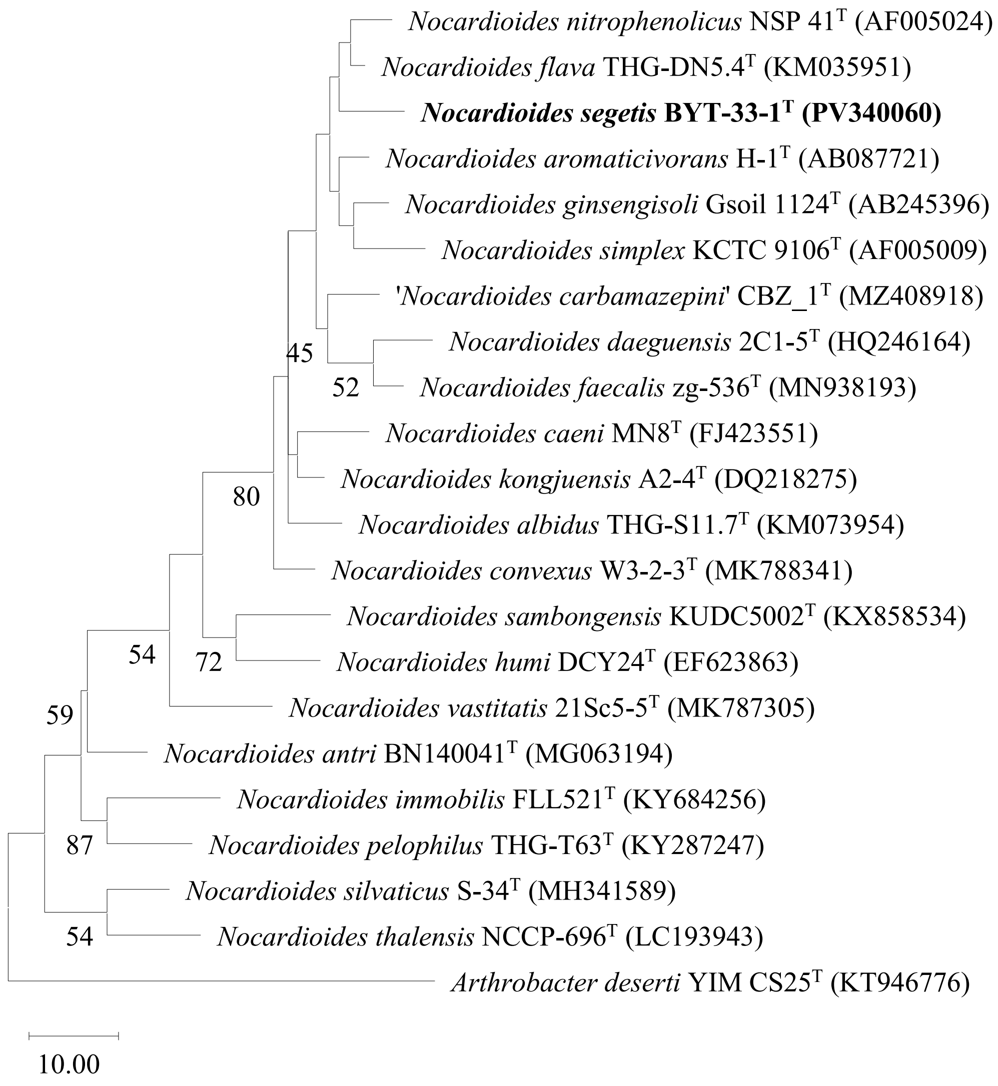

Fig. S2. Neighbor-joining phylogenetic tree of strain BYT-33-1^T and its relatives based on the comparison of the 16S rRNA gene sequences. Genbank accession numbers were given in parentheses.

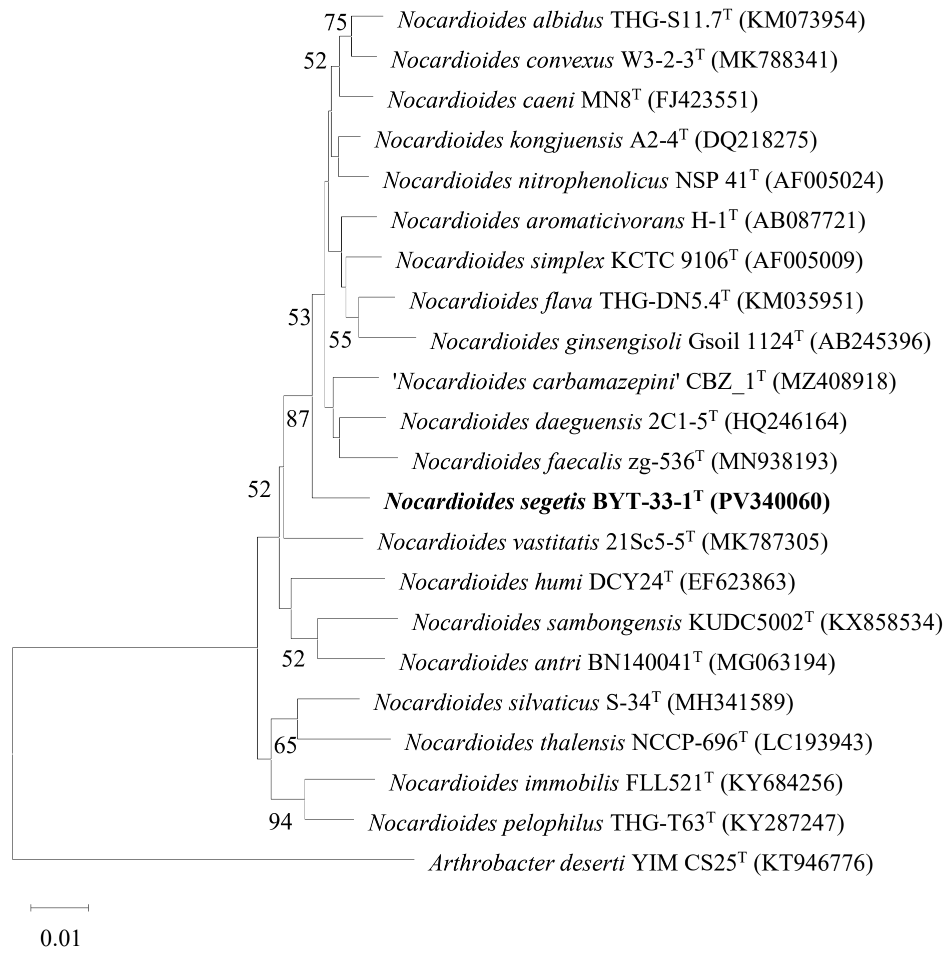

Fig. S3. Genomics information of BYT-33-1^{T}.

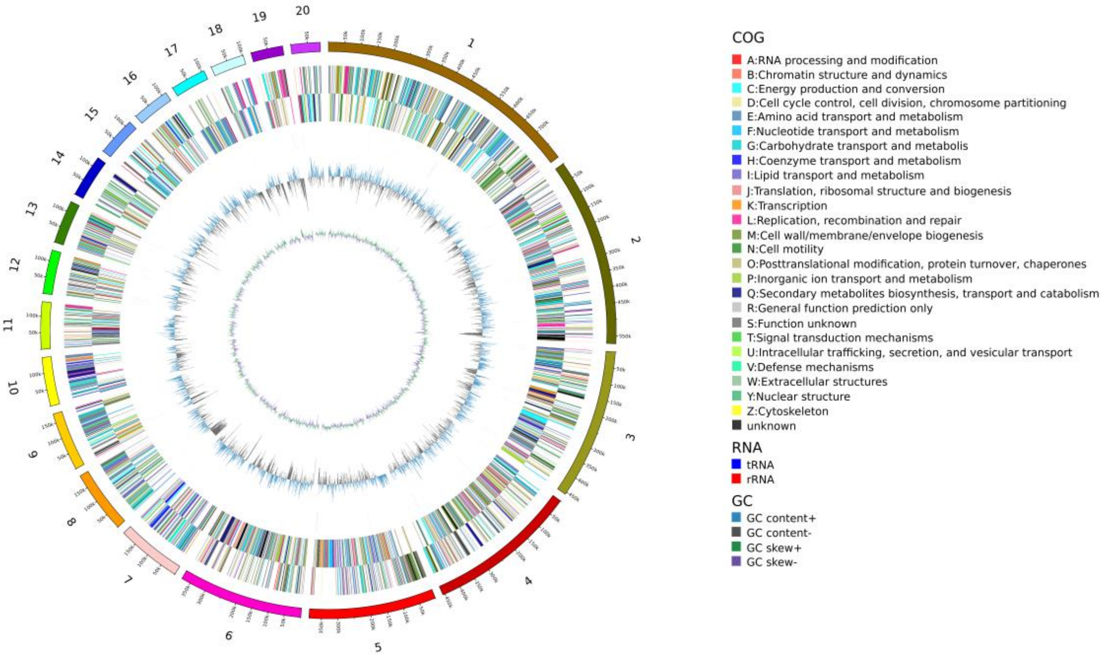

Fig. S4. Function classification of strain BYT-33-1^{T}.

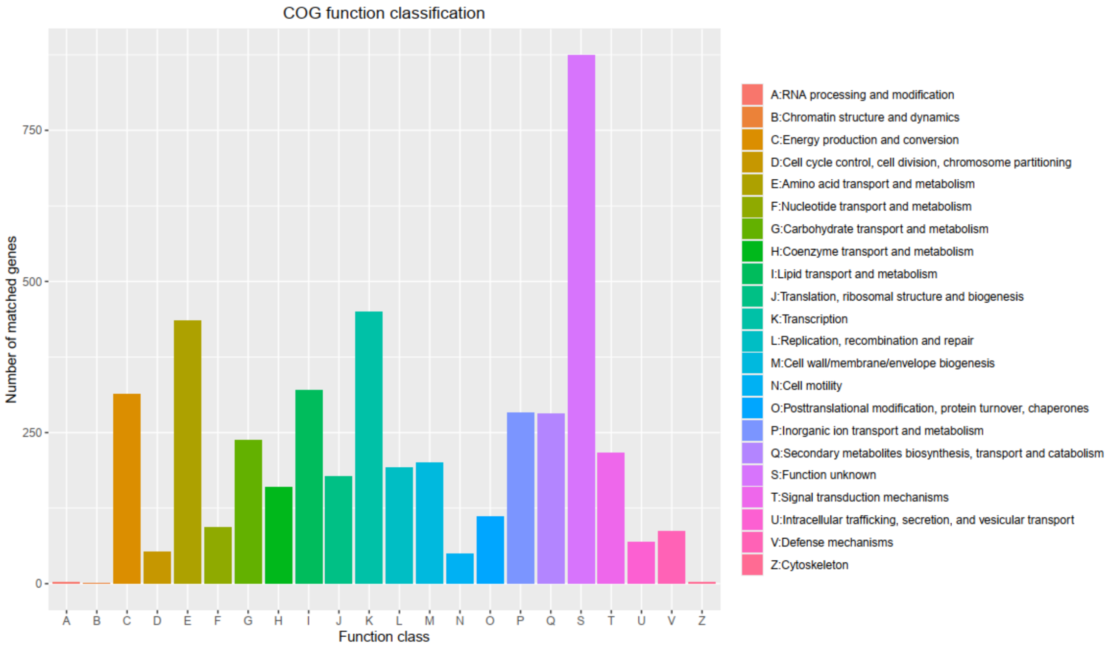

Fig. S5. Transmission electron micrograph of cell of strain BYT-33-1^T. Bar, 0.5 μm. The strain was incubated on LB medium at 30 °C for 48 h.

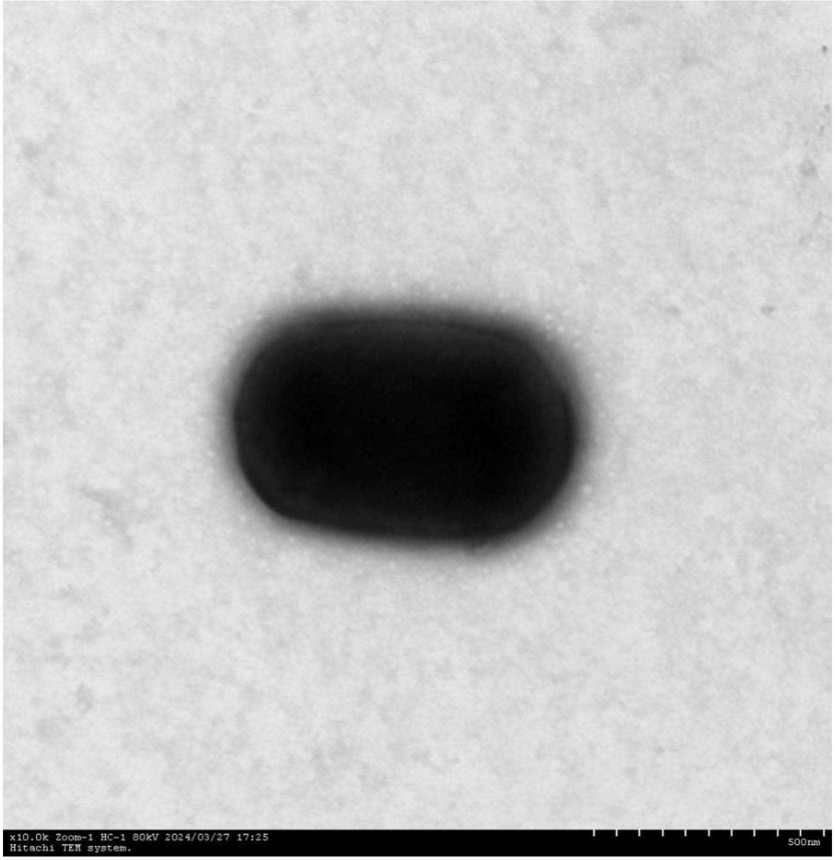

Fig. S6. GC-FID chromatogram of residual hexadecane after incubation with strain BYT-33-1^{T}.

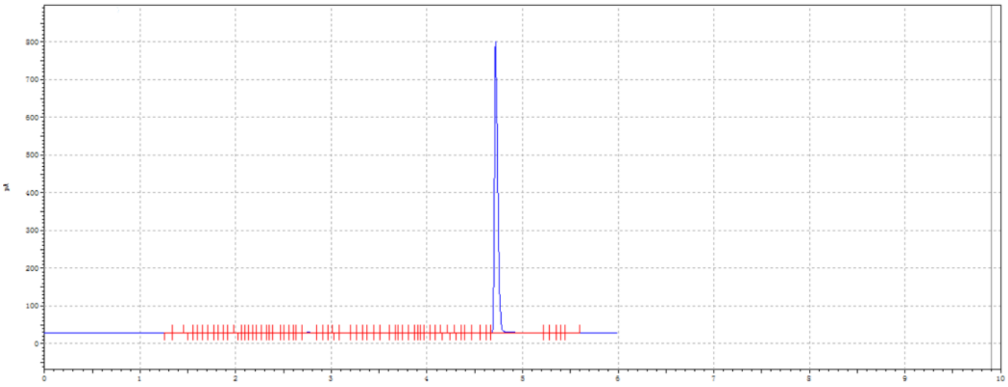

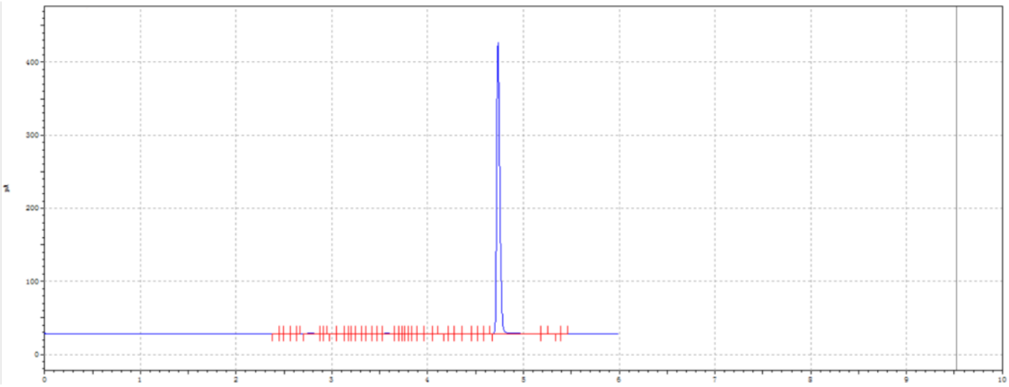

Fig. S7. DPG, diphosphatidylglycerol; PG, phosphatidylglycerol; AL, unidentified aminolipid; PL1-3, unidentified phospholipid. (a). molybdatophosphoric acid; (b). molybdenum blue; (c). ninhydrin.

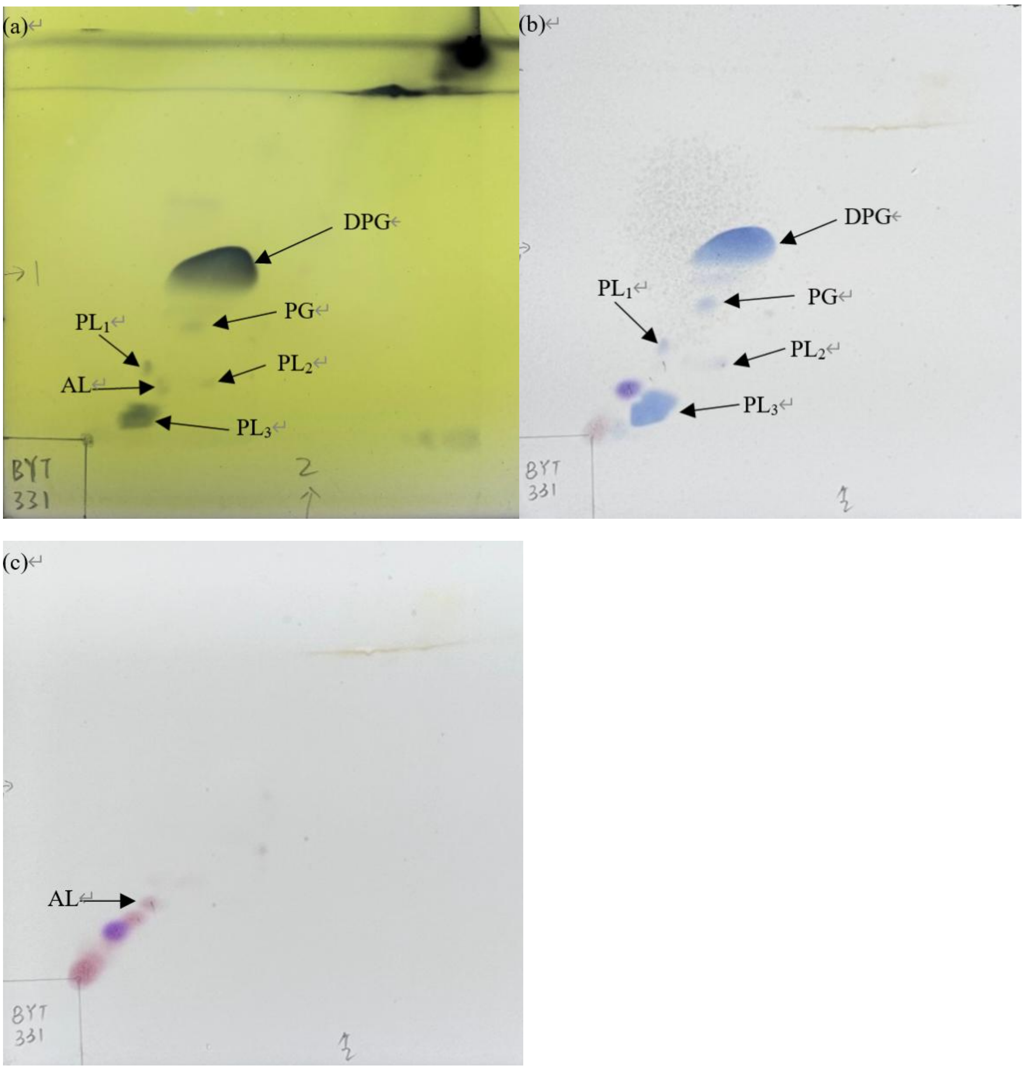

Supplement: Uncited Supplementary Material 1. [file ijsem-76-07160-s001.pdf]
